# Somatotype characteristics of normal-weight and obese women among different metabolic subtypes

**DOI:** 10.1590/2359-3997000000159

**Published:** 2016-01-01

**Authors:** Biljana Srdić Galić, Tatjana Pavlica, Mirjana Udicki, Edita Stokić, Milena Mikalački, Darinka Korovljev, Nebojša Čokorilo, Zorka Drvendžija, Dragan Adamović

**Affiliations:** 1 University of Novi Sad Faculty of Medicine Department of Anatomy Novi Sad Serbia University of Novi Sad, Faculty of Medicine, Department of Anatomy, Novi Sad, Serbia; 2 University of Novi Sad Faculty of Sciences Department of Biology and Ecology Novi Sad Serbia University of Novi Sad, Faculty of Sciences, Department of Biology and Ecology, Novi Sad, Serbia; 3 University of Novi Sad Faculty of Medicine Department of Endocrinology, Diabetes and Metabolic Disorders Novi Sad Serbia University of Novi Sad, Faculty of Medicine, Department of Endocrinology, Diabetes and Metabolic Disorders, Novi Sad, Serbia; 4 University of Novi Sad Faculty of Sport and Physical Education Novi Sad Serbia University of Novi Sad, Faculty of Sport and Physical Education, Novi Sad, Serbia

**Keywords:** Somatotype, obesity, metabolic syndrome, metabolically healthy obese, metabolically obese normal-weight

## Abstract

**Background:**

Obesity is a well known risk factor for the development of metabolic abnormalities. However, some obese people are healthy and on the other hand some people with normal weight have adverse metabolic profile, therefore it can be assumed that there is a difference in physical characteristics amongst these people. The aim of this study was to establish whether there are somatotype differences between metabolically healthy and metabolically obese women who are obese or of normal weight.

**Subjects and methods:**

Study included 230 women aged 44.76 ± 11.21y. Metabolic status was assessed according to IDF criteria, while somatotype was obtained using Heath & Carter method.

**Results:**

Significant somatotype differences were observed in the group of women with normal-weight: metabolically healthy women had significantly lower endomorphy, mesomorphy and higher ectomorphy compared to metabolically obese normal-weight women (5.84-3.97-2.21 vs. 8.69-6.47-0.65). Metabolically healthy obese women had lower values of endomorphy and mesomorphy and higher values of ectomorphy compared to ‘at risk’ obese women but the differences were not statistically significant (7.59-5.76-0.63 vs. 8.51-6.58-0.5). Ectomorphy was shown as an important determinant of the favorable metabolic profile (cutoff point was 0.80).

**Conclusion:**

We concluded that, in addition to fat mass, metabolic profile could be predicted by the structure of lean body mass, and in particular by body linearity.

## INTRODUCTION

Somatotyping provides the quantitative description of the human physique. The most widely used somatotype method was introduced by Heath & Carter; it is expressed in three components (endomorphy, mesomorphy and ectomorphy) that empirically define different aspects of the body composition: degree of fatness, musculoskeletal development and the linearity of the body (1). Each individual is a unique combination of all the above three components in different proportion. Endomorphy, mesomorphy and ectomorphy correspond with the three primary germ cell layers that give rise to the specific sets of tissues that define body composition.

Obesity, especially central (truncal) type, has been proven to be an independent risk factor for the development of cardiovascular and metabolic disturbances. However, some phenotypically obese individuals have normal metabolic profile. Some studies indicate that 10-25% of obese individuals are actually metabolically healthy (2-4). On the other hand, it has been estimated that 13-18% of normal-weight individuals have abnormal metabolic profile (5,6). The mechanisms underlying the metabolic disturbances in metabolically obese normal weight subjects, as well as those that prevent the development of metabolic abnormalities in metabolically healthy obese subjects, are poorly understood. It is assumed that the muscle metabolic capacity and also the ability to store fat in the subcutaneous adipose tissue depots instead of in visceral depots could be of great importance in understanding these phenomena (4,7). This would imply certain somototype differences between metabolically healthy and metabolically obese individuals of the same nutrition level. The purpose of this study was to analyse somatotype in normal-weight and obese women with respect to their metabolic profile.

## SUBJECTS AND METHODS

Study group involved 230 women aged 22 to 76 years (average age: 44.76 ± 11.21y) who voluntarily participated in the study. This investigation was taken as a part of a larger cross-sectional population study of the prevalence of obesity and cardiovascular risk factors among adult population living in the urban and rural areas of Vojvodina province situated in the northern part of Serbia. Vojvodina represents the most demographically diverse region of Serbia with more than 25 ethnic groups (most prominent ethnic groups are Serbs and Hungarians). Participants were invited to participate in the study via local media, pamphlets and social networks. Participants underwent thorough evaluation, including medical and family history, physical examination and blood biochemistry by health professionals; all the tests included were free of charge for all participants. Candidates with any of the following conditions were excluded from the study: history or evidence of cardiovascular diseases, diabetes, malignancies, chronic liver disease, using steroids, hormone displacement therapy, or medication that could affect body composition, cardiovascular function or metabolism, pregnancy, currently breastfeeding, and large body mass fluctuations in the last 6 months. Participants who had missing data or presented difficulties with measuring were also excluded. The study was carried out pursuant to the Declaration of Helsinki. In order to assess somatotype, nutrition level and metabolic profile all subjects underwent anthropometric measurements, blood pressure measurements and biochemical analyses.

Body mass was obtained through body composition assessment using the bioelectrical impedance analysis (Tanita TBF-310 bioimpedance analyzer, Tanita Corporation, Tokyo, Japan). Body height was measured to the nearest 0.1 cm using GPM anthropometer (Sieber&Hegner, Zürich, Switzerland). Body girths (flexed and tensed upper arm girth, waist girth and calf girth) were measured using Holtain flexible but non-stretchable tape (Holtain Ltd, Croswell, UK) to the nearest 0.1 cm. Upper arm girth was measured as the maximal girth of the upper arm with flexed and tensed elbow. Calf girth was measured as the greatest girth of the calf. Waist circumference was measured at the level midway between the lowest point of the rib margin and the highest point of the iliac crest. Skinfold thicknesses (triceps, subscapular, supraspinale and medial calf) were measured using Harpenden caliper (Holtain Ltd, Croswell, UK) to the nearest 0.2 mm. Triceps skinfold thickness was measured in the vertical direction at the level halfway between the acromion and olecranon. Subscapular skinfold thickness was measured below the inferior angle of the scapula in an oblique direction downwards and laterally at 45 degrees. Supraspinale skinfold thickness was measured above the anterior superior iliac spine on a line to the anterior axillary border and on a diagonal line going downwards and medially at 45 degrees. Medial calf skinfold thickness was measured in the vertical direction on the medial side of the leg, at the level of the maximum calf girth. Biepicondylar humeral and femoral breadth were measured using Holtain bicondylar caliper (Holtain Ltd, Croswell, UK) to the nearest 0.1 cm, between lateral and medial epicondyles of the humerus and femur, compressing the subcutaneous tissue.

Somatotype was assessed using Heath & Carter method and nutritional status was assessed according to the body mass index (BMI) standards: normal-weight 18.5-24.9 kg/m^2^, overweight and obesity ≥ 25 kg/m^2^ (1,8).

Biochemical factors including plasma glucose, triglycerides and HDL-cholesterol were determined in overnight fasting blood sample. Glucose was analyzed using Dialab glucose GOD PAP method, tryglicerides were analyzed using an enzymatic method and HDL-cholesterol was analysed using magnesium chloride/phosphotungstate precipitation technique. Systolic and diastolic blood pressure were measured in the morning using a Riva-Rocci sphygmomanometer. Using the IDF criteria subjects were defined as having the metabolic syndrome if they had central obesity (defined as waist circumference of ≥ 80 cm) plus any two of the following four factors: blood pressure of ≥ 130/85 mmHg, glucose of ≥ 5.6 mmol/L, high density lipoprotein cholesterol (HDL-cholesterol) of < 1.29 mmol/L and triglyceride of ≥ 1.7 mmol/L (9).

According to BMI and metabolic profile the examined group was subdivided into four subgroups: metabolically healthy normal-weght (BMI < 25 kg/m^2^ and the absence of metabolic syndrome), metabolically obese normal-weight (BMI < 25 kg/m^2 ^and the presence of metabolic syndrome), metabolically healthy obese (BMI ≥ 25 kg/m^2^ and the absence of metabolic syndrome) and ‘at risk’ obese (BMI ≥ 25 kg/m^2^ and the presence of metabolic syndrome).

Results are presented as mean ± standard deviation (SD) and percent. The one-way analysis of variance (ANOVA) with the Bonferroni post-hoc method was used to determine whether there are any significant differences between the means of the subgroups. The discrimination abilities (accuracy) of endomorphy, mesomorphy and ectomorphy in the prediction of metabolic syndrome were assessed with the area under the receiver-operating characteristic (ROC) curve. The statistical program used for the calculations was SPSS Statistics 17.0 (IBM SPSS, Chicago, IL).

## RESULTS

Characteristics of the examined subjects are presented in the Table 1. According to the BMI values 35.65% of women were overweight or obese while 64.35% were of normal-weight; 9.46% of normal-weight subjects were metabolically obese, while 13.41% of obese subjects were metabolically healthy.

**Table 1:** Physical and metabolic characteristics of examined women

	Mean ± SD
Body height (cm)	164.76 ± 11.21
Body mass (kg)	69.33 ± 12.79
Body mass index (kg/m^2^)	27.97 ± 6.48
Flexed upper arm girth (cm)	29.82 ± 3.58
Waist girth (cm)	81.60 ± 12.45
Calf girth (cm)	36.79 ± 3.20
Triceps skinfold (mm)	24.53 ± 8.41
Subscapular skinfold (mm)	23.18 ± 10.88
Supraspinale skinfold (mm)	24.16 ± 9.30
Medial calf skinfold (mm)	25.47 ± 9.48
Biepicondylar humerus breadth (cm)	6.47 ± 0.68
Biepicondylar femur breadth (cm)	9.45 ± 1.10
Systolic blood pressure (cm)	116.19 ± 16.65
Diastolic blood pressure (cm)	75.07 ± 10.46
Glucose (mmol/L)	4.67 ± 0.67
HDL-cholesterol (mmol/L)	1.52 ± 0.69
Tryglicerides (mmol/L)	1.33 ± 0.69

Somatotype analysis showed significantly higher values of endomorphy and mesomorphy and lower values of ectomorphy in the overweight and obese compared to normal-weight women (Table 2). Considering the metabolic profile, metabolically obese subjects had higher values of endomorphy and mesomorphy and lower values of ectomorphy compared to the metabolically healthy counterparts (Table 3). However, significant differences between metabolically healthy and metabolically obese individuals were found only in the group of normal-weight women. Metabolically healthy normal-weight women had significantly lower endomorphy and mesomorphy and higher ectomorphy compared to the somatotype of all the other subgroups. Somatotype of metabolically obese normal-weight women did not differ significantly from the somatotype of ‘at risk’ obese women, but endomorphy of metabolically obese normal-weight women was significantly higher compared to the metabolically healthy obese women.

**Table 2:** Somatotype in normal-weight and overweight and obese women

	Normal-weight	Overweight and obese	ANOVA
Endomorphy	5.52 ± 1.23*	7.55 ± 1.48	0.000
Mesomorphy	3.66 ± 1.09*	5.65 ± 2.07	0.000
Ectomorphy	2.34 ± 0.93*	0.96 ± 0.97	0.000

* Significantly different from overweight and obese subjects.

**Table 3:** Somatotype in normal-weight and overweight or obese women of different metabolic profiles

Somatotype	Normal-weight			Overweight/obese		ANOVA
	Metabolically healthy	Metabolically obese		Metabolically healthy	Metabolically obese	
Endomorphy	5.84 ± 1.54*	8.69 ± 0.84^†^		7.59 ± 1.13	8.51 ± 0.81	0.000
Mesomorphy	3.97 ± 1.44*	6.47 ± 1.79		5.76 ± 2.12	6.58 ± 1.93	0.000
Ectomorphy	2.21 ± 1.12*	0.65 ± 0.32		0.63 ± 0.23	0.5	0.000

* Significantly different from metabolically obese normal-weight, metabolically healthy obese and ‘at risk’ obese subjects; † Significantly different from metabolically healthy obese subjects.

**Figure 1:**
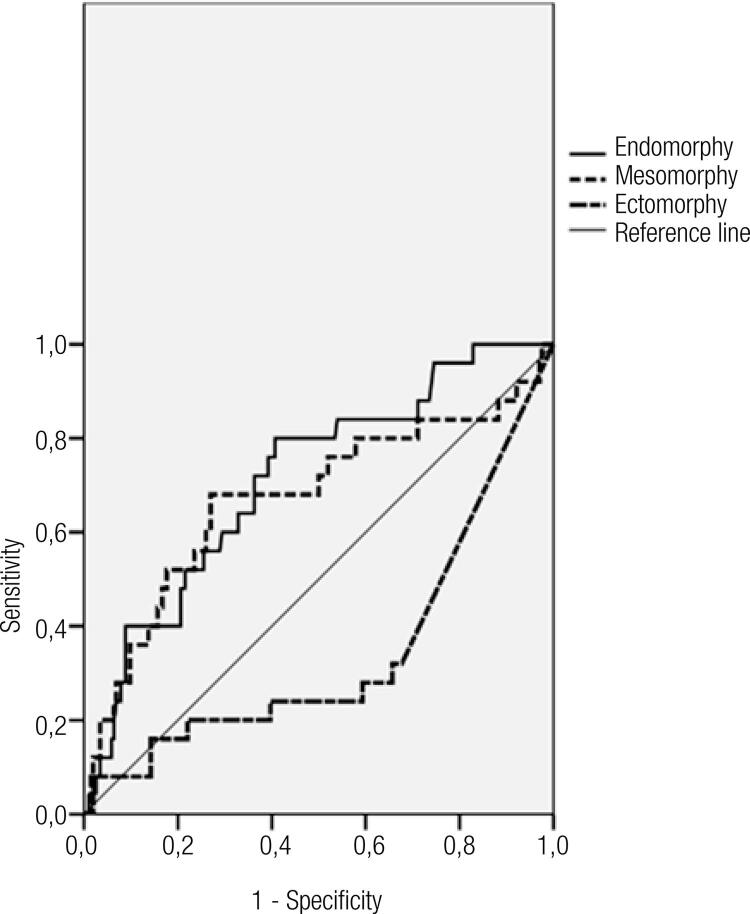
Receiver operating characteristic (ROC) curve for endomorphy, mesomorphy and ectomorphy in a prediction of metabolic syndrome.

Receiver operational characteristic (ROC) curve analysis revealed the maximum predictive value of endomorphy for metabolic syndrome (AUC: 0.713). The AUC of mesomorphy was 0.673 in identifying metabolic syndrome. Ectomorphy was shown as a best predictor of the favorable metabolic profile (AUC: 0.658) (Table 4, Figure 1).

**Table 4:** Performance of somatotype in the prediction of metabolic syndrome

	AUC (95% CI)	Cut-off	Sensitivity	Specificity
Endomorphy	0.713	6.89	0.800	0.407
Mesomorphy	0.673	5.35	0.680	0.270
Ectomorphy	0.342	0.80	0.280	0.657

## DISCUSSION

Our study aimed at revealing somatotype differences between different metabolic subtypes of obesity. The obtained results imply the important role of the non- adipose components, presented by mesomorphy and ectomorphy, in the distinction between healthy and risky metabolic profile.

Somatotype describes different aspects of body composition. It is used in the assessment of the changes in physique during growth, ageing and physical activity. However, some studies showed that it could be used in the prediction of certain diseases. According to Koleva and cols., mesomorphic endomorphs tend to suffer from digestive system disorders, neurosis, or lumbo-sacral radiculitis (10). The same study showed that individuals of both genders with higher endomorphy and mesomorphy and lower ectomorphy more frequently suffer from arterial hypertension and liver disease. Higher values of endomorphy were reported in metabolic syndrome, type 2 diabetes, hypertension and breast cancer (10-13). Baltadjiev found that the diabetic individuals mostly present with endomorphic mesomorph or mesomorph-endomorph somatotype, pointing also to age-, gender-, and population dependent somatotype differences (14,15). Our results showed higher values of endomorphy and mesomorphy and lower values of ectomorphy in overweight subjects. However, our results reveal some differences between metabolically healthy and metabolically obese women of the same nutrition level. In both, normal-weight and obese women metabolically obese subjects had higher endomorphy and mesomorphy and lower ectomorphy than the metabolically healthy subjects. Somatotype of metabolically healthy normal-weight women was completely distinct showing significantly lower endomorphy and mesomorphy and higher ectomorphy comparing to the other subgroups. Somatotype of the metabolically obese normal-weight women was similar to the somatotype of those who were overweight and obese, with even significantly higher values of endomorphy compared to the metabolically healthy obese women. The role of adipose tissue in the development of the cardiovascular and metabolic disorders has been well recognized, so the results obtained for endomorphy were not unexpected.

Metabolically obese normal-weight individuals are known to demonstrate metabolic disturbances in spite of normal values of BMI. Several studies showed higher body fat (especially visceral depot) and low lean mass in the metabolically obese normal-weight individuals (6,16-18). According to our results, mesomorphy was higher in metabolically obese women who were both, normal-weight and overweight. In order to explain obtained results the storage capacity of adipose tissue and the muscle metabolic capacity should be considered. It is assumed that the metabolic risk is determined by the capability of subcutaneous adipose tissue to store fat. Fat is initially stored in the subcutaneous adipose tissue, but once the capacity of subcutaneous adipose tissue is reached, storage shifts to visceral depots and ectopic non-adipose sites, including skeletal muscles (19,20). Higher mesomorphy thus could be the result of the deposition of the ectopic fat in skeletal muscles that causes larger girths of extremities. Additionaly, mesomorphy reflects the muscular mass but it also contains some measures of peripheral fat which could explain higher mesomorphy in the metabolically obese women registered in our study.

Higher ectomorphy in metabolically healthy individuals implies the importance of the body linearity. Several studies showed the inverse correlation between body height and cardiometabolic risk (21,22). Our previous results also showed that metabolically healthy obese women are significantly higher than the ‘at risk’ obese ones (23). Some authors explain this phenomenon by the fetal undernutrition which causes the tissue reprogramming in a way that determines further development of insulin resistance and atherosclerosis and changes of postnatal body composition (24). This could be in line with the embryological aspect of different components of somatotype.

In analyzing the capability of somatotype to predict metabolic risk, endomorphy and mesomorphy were shown as better predictors of metabolic syndrome (cut-offs were 6.89 and 5.35, respectively) while ectomorphy was the best predictor of the favorable metabolic profile (cut-off was 0.8).

In conclusion, our results clearly show somatotype differences between metabolically healthy and metabolically obese normal-weight women. Endomorphy was the best predictor of metabolic syndrome. Higher mesomorphy in metabolically obese women appears to be controversial since it basically reflects muscularity. An important finding was the higher ectomorphy in metabolically healthy individuals which highlights the protective role of higher body linearity in the development of metabolic syndrome. Despite the minor clinical relevance of the somatotyping it could help in the explanation of the underlying mechanisms of metabolically healthy obesity and of metabolic abnormalities in normal-weight individuals. Concerning embryonic aspect of the somatotype theory our results may indicate that the susceptibility to metabolic syndrome could be determined by the prenatal environment. Finally, our results support the close relationship between body constitution and metabolic phenotype. Defining optimal somatotype could help in differentiation between metabolically healthy and metabolically obese individuals of the same nutrition level.
